# PSMA Theranostics: Review of the Current Status of PSMA-Targeted Imaging and Radioligand Therapy

**DOI:** 10.3390/cancers12061367

**Published:** 2020-05-26

**Authors:** Wallace Jones, Kelly Griffiths, Pedro C. Barata, Channing J. Paller

**Affiliations:** 1 Department of Internal Medicine, Tulane University School of Medicine, New Orleans, LA 70112, USA; wjones20@tulane.edu; 2 Sidney Kimmel Comprehensive Cancer Center, Johns Hopkins University, Baltimore, MD 21231, USA; kgriff43@jhmi.edu; 3 Department of Hematology-Oncology, Tulane University School of Medicine, New Orleans, LA 70112, USA

**Keywords:** prostate specific membrane antigen, theranostics, prostate cancer

## Abstract

Prostate-specific membrane antigen (PSMA) has been the subject of extensive investigation in the past two decades as a promising molecular target for prostate cancer (PCa). Its appealing molecular features have enabled the development of a novel diagnostic and therapeutic—thus “theranostic”—approach to PCa. There is now substantial evidence of the high sensitivity of PSMA-targeted imaging for PCa lesions and growing evidence of the therapeutic efficacy of PSMA radioligand therapy for metastatic castration-resistant prostate cancer. This article presents a broad overview of the current status of PSMA theranostics, including current evidence, potential clinical impact, and active areas of research.

## 1. Introduction

Also known as folate hydrolase I or glutamate carboxypeptidase II, prostate-specific membrane antigen (PSMA) is a type II, 750 amino acid transmembrane protein. In benign prostatic cells, it is localized to the cytoplasmic and apical side of the prostate epithelium. As malignant transformation occurs, PSMA is transferred from the cytoplasm to the luminal surface of the prostatic ducts, where it presents a large extracellular domain to ligands [1]. The biological function of PSMA remains unclear, but it is hypothesized to have a transport function because PSMA ligands are internalized through endocytosis.

PSMA ligand internalization theoretically enables specificity of synthetic PSMA radioligands for malignant prostatic tissue. Furthermore, research suggests a 100- to 1000-fold increase in PSMA expression in prostatic adenocarcinoma vs. benign prostatic tissue [2,3]. Although there is an increasing understanding of inter- and intra-patient heterogeneity of expression, PSMA expression generally increases with tumor dedifferentiation and in metastatic castration-resistant prostate cancer (mCRPC). Neuroendocrine PCa may be an exception to this rule, as case reports suggest that the PSMA gene (*FOLH1*) may be suppressed in neuroendocrine prostate cancer (PCa) [4].

Despite its name, PSMA is expressed in various benign and neoplastic tissues. Histopathological studies have confirmed PSMA expression in salivary glands, duodenal mucosa, proximal renal tubular cells, and neuroendocrine cells in the colonic crypts [5]. However, PSMA expression is substantially lower in these tissues than in PCa lesions [6,7]. Studies have observed PSMA radiotracer uptake in various non-prostatic tissues. High uptake of the novel PSMA radiotracer ^64^copper (^64^Cu)-PSMA has been observed in salivary glands, kidneys, and the liver [8]. High uptake of ^68^gallium (^68^Ga)-PSMA-11 has been observed in these tissues, as well as in the bladder, duodenum, small intestine, spleen, lacrimal glands, and various mucosal tissues [9,10,11]. However, there is no radiotracer accumulation in common sites of PCa metastasis, such as regional and distant lymph nodes and bone, in the absence of metastasis.

## 2. PSMA-Targeted Imaging Modalities

PSMA-targeted imaging is appealing, given the inadequate sensitivity of conventional imaging technologies for low-volume PCa lesions and thus their suboptimal performance for detection of early biochemically recurrent prostate cancer (BRPC) and occult metastatic PCa. For instance, the detection rates of multiparametric magnetic resonance imaging (MRI) at prostate-specific antigen (PSA) levels less than 0.4 ng/mL are low; computed tomography (CT) and bone scan rarely detect the anatomic site of recurrence at PSA values amenable to salvage therapy. Similarly, CT and MRI have modest sensitivity (42% and 39%, respectively) and specificity (82% and 82%) for detection of lymphatic disease [12]. Novel positron-emission tomography (PET) radiotracers targeting PSMA, including fluorine-18 (^18^F)- and ^68^Ga-labeled compounds, have demonstrated significantly higher detection rates.

Anti-PSMA antibodies were the earliest synthesized PSMA ligands and were found to have good tumor detection rates but limited clinical potential. Monoclonal PSMA antibodies are produced by immunizing mice with a peptide corresponding to PSMA extracellular residues [13]. In the initial clinical trial of the monoclonal antibody muJ591, which was linked to iodine-131 (^131^I), muJ591 demonstrated excellent targeting of PCa metastases [14]. However, the clinical utility of J591 and other PSMA antibodies is limited by low tumor penetrability. Additionally, due to the relatively long half-lives of anti-PSMA antibodies, there is a long delay between injection and imaging, which leads to high accumulation in inflammatory tissue and substantial radiation exposure.

The synthesis of small-molecule PSMA inhibitors marked a significant advance in PSMA theranostics. Compared to anti-PSMA antibodies, small-molecule PSMA inhibitors have similarly accurate localization of PCa lesions but faster tumor uptake and more rapid excretion, which reduces radiation exposure. A variety of inhibitors have now been synthesized, but studies have primarily used ^68^Ga-labeled radiotracers, the development of which was considered a breakthrough due to their excellent characteristics, such as high tumor contrast. ^68^Gallium-PSMA-11 (^68^Ga-PSMA-11 or ^68^Ga-PSMA-HBED-CC) is the most commonly used compound. PSMA ligands may be used for single-photon-emission computed tomography (SPECT), PET/MRI, and PET/CT, but most studies have utilized PSMA-PET/CT.

## 3. Uses and Impact of PSMA-Targeted Imaging

### 3.1. Efficacy of PSMA-Targeted Imaging for Localized PCa

PSMA-targeted imaging research has primarily evaluated diagnostic efficacy for BRPC, and results suggest a high positive predictive value (PPV) and sensitivity, even at low PSA levels. In a recent systematic review of 43 studies and 5113 patients with BRPC and no known metastatic disease, the pooled detection rates for PSMA radiotracers in patients with BRPC after definitive therapy and a PSA level of <0.5, 0.5 to 0.9 ng/mL, 1.0 to 1.9 ng/mL, and ≥2 ng/mL were 45%, 61%, 78%, and 94%, respectively [15]. Importantly, the rate of detection at very low PSA values (less than 0.5 ng/mL) was significantly higher than that of conventional imaging. In the first prospective trial of ^68^Ga-PSMA-11 PET/CT for BRPC, which included 635 patients, detection rates were 38% for PSA levels of <0.5 ng/mL (*n* = 136), 57% for 0.5 to <1.0 ng/mL (*n* = 79), 84% for 1.0 to < 2.0 ng/mL (*n* = 89), 86% for 2.0 to <5.0 ng/mL (*n* = 158), and 97% for ≥ 5.0 ng/mL (*n* = 173) [16]. Investigators reported high a PPV for the detection of BRPC (0.84 by histopathological validation (95% CI: 0.75–0.90) and high inter-reader agreement (Fleiss κ, 0.65–0.78).

Head-to-head studies of PSMA-targeted imaging and conventional imaging for detection of BRPC suggest superior performance of PSMA-targeted imaging. A cohort study comparing ^68^Ga-PSMA-11 with ^18^F-fluoromethylcholine that included 38 patients with BRPC and PSA levels ranging from 0 to 0.5 ng/mL, 0.5 to 2.0 ng/mL, and above 2.0 ng/mL, reported detection rates of 50% vs. 12.5%, 69% vs. 31%, and 86% vs. 57%, respectively [17]. In a study in which ^68^Ga-PSMA-11 PET/CT was only performed in patients with negative ^18^F-choline-PET/CT scans, ^68^Ga-PSMA-11 PET/CT identified sites of recurrent disease in 43.8% of patients [18]. In the preliminary analysis of an ongoing prospective study of ^68^Ga-PSMA-11 and ^18^F-fluciclovine that included 50 patients with BRPC after primary prostatectomy, ^68^Ga-PSMA-11 demonstrated consistently higher detection rates than ^18^F-Fluciclovine in all anatomic regions: prostate bed (20% vs. 12%, respectively), pelvic nodes (37% vs. 14%), extra-pelvic nodes (8% vs. 2%), skeleton (8% vs. 2%), and visceral organs (6% vs. 2%) [19]. In a study comparing ^68^Ga-PSMA-11 with ^18^F-Fluciclovine, Pernthaler et al. observed almost equivalent detection rates for distant metastases, but poorer performance of ^68^Ga-PSMA-11 in detection of curable localized disease close to the bladder, possibly due to radiotracer accumulation [20].

Multiple novel PSMA radioligands have demonstrated sensitivity for BRPC comparable to ^68^Ga-PSMA-11, as well as potential advantages. Fluorine-18 (^18^F)-labeled PSMA-ligands have shown comparable sensitivity for BRPC lesions and enhanced image quality, suggesting potentially improved detection of small metastases. In a cohort study of 248 patients, Wondergem et al. reported comparable efficacy of fluorine-18 DCFPyL (^18^F-DCFPyL) to ^68^Ga-PSMA-11 and potentially increased efficacy for patients with PSA < 2.0 [21]. ^18^F-PSMA-1007 has been found to have diagnostic accuracy comparable to ^68^Ga-PSMA-11 for detection of BRPC and is only minimally excreted in the urinary tract, suggesting a potential advantage for pelvic imaging [22,23]. Additionally, logistical concerns surrounding ^68^Ga-PSMA-11 PET/CT may be mitigated by novel radiotracers. ^68^Ga-PSMA-11 PET/CT requires an onsite ^68^germanium (^68^Ge)/^68^Ga generator, and, if available, ^68^Ge/^68^Ga generators may also have limited production. The longer half-life of ^18^F-labeled compounds may facilitate production and permit longer-distance delivery. Novel radiotracers may also be useful to PET/CT centers lacking a ^68^Ge/^68^Ga generator.

### 3.2. PSMA-Targeted Imaging of Metastatic Disease

PSMA-targeted imaging has demonstrated higher sensitivity for the detection of lymph node metastases (LNM) than conventional imaging. In a cohort of 20 patients, ^68^Ga-PSMA-11 PET/CT demonstrated higher sensitivity and specificity than MRI but comparable efficacy to DW-MRI [24]. In a study of 38 patients planned to undergo salvage lymphadenectomy, ^68^Ga-PSMA-11 PET/CT had significantly higher negative predictive value (NPV) and accuracy for detection of LNM than ^18^F-fluoroethylcholine PET/CT [25]. In a study of 65 patients who underwent ^68^Ga-PSMA-11 scanning prior to salvage lymph node dissection following biochemical recurrence, Abufaraj et al. reported sensitivity ranging from 72% to 100% and specificity ranging from 96% to 100% [26]. In a retrospective study with a larger cohort of 130 patients with intermediate-to-high risk PCa staged preoperatively with ^68^Ga-PSMA-PET/CT, Maurer et al. reported sensitivity and specificity of 99.1% and 95.2%, respectively, outperforming CT and MRI [27]. Region-specific PPV and NPV in this study ranged from 95% to 100% and 93% to 100%, respectively. In the first prospective study of 23 patients, ^64^Cu-PSMA PET/CT demonstrated similar efficacy for detection of LNM, with a reported sensitivity of 87.5% and specificity of 100% [28]. Despite promising results, a negative correlation between lymph node size and diagnostic accuracy of PSMA-PET/CT has been described, raising concerns about low sensitivity for micrometastatic nodal tumor deposits [29].

Although few studies have investigated PSMA localization of bone metastases, preliminary research suggests that PSMA-PET/CT may be superior to bone scanning. A recent systematic review of 31 case series suggested that ^68^Ga-PSMA-PET/CT identified more lesions than bone scans but noted that the large majority of studies were retrospective and did not include a reference standard [30]. A study of 415 patients who underwent ^68^Ga-PSMA-PET/CT observed detection rates for bone metastasis of 48.3%, 52.6%, 74.4%, 79.6%, and 93.9% for PSA values of < 0.2 ng/mL, 0.2–0.5 ng/mL, 0.5–1 ng/mL, 1–2 ng/mL, and >2 ng/mL, respectively [31]. PSMA-PET/CT detected 258 suspicious regions, 255 of which were metastatic and 3 of which were equivocal, whereas bone scanning detected only 223 suspicious regions, 203 of which were metastatic and 20 of which were equivocal.

Variables that may influence the performance of PSMA-targeted imaging include PSA, Gleason score, and the presence of ongoing androgen-deprivation therapy (ADT). As expected, there is a strong correlation between lesion-detection rate and increasing PSA level. However, a rising PSA level does not always correlate with an increased tumor-detection rate, as patients with PSA levels above 10 ng/mL have been noted to have negative ^68^Ga-PSMA-11 PET/CT scans [32]. Possible explanations for this observation include tumor location adjacent to the urinary bladder and inter-patient heterogeneity in PSMA expression. Studies have reported conflicting results with regard to Gleason score and probability of a pathological scan [33]. Regarding ADT, preclinical studies suggest that ADT increases expression of PSMA in PCa cells [34,35,36,37]. The effect of ongoing ADT on ^68^Ga-PSMA-11 PET/CT efficacy, however, is unclear, with studies reporting either a positive correlation or no significant association [32,33,38,39].

PSMA expression in various benign tissues and non-prostatic malignancies has led to concerns about the specificity of PSMA-targeted imaging for metastatic disease. Neoplastic tissues with PSMA expression have been described, including transitional cell carcinoma, hepatocellular carcinoma, renal cell carcinoma, and colorectal carcinoma. False positives in benign conditions are also increasingly noted, such as ^68^Ga-PSMA-11 uptake in sarcoidosis and Paget’s disease and ^64^Cu-PSMA uptake in pneumonitis [10,40,41]. However, PSMA-targeted imaging has demonstrated higher specificity for PCa than conventional imaging. Moreover, as understanding of the physiological distribution of PSMA improves, unusual sites of tracer avidity are less likely to lead to false positive interpretation.

### 3.3. Clinical Impact of and Future Directions for PSMA-Targeted Imaging

Pending results from ongoing prospective trials (Table 1), PSMA-targeted imaging may eventually play multiple roles in the management of PCa. Given the substantial evidence of higher sensitivity of PSMA-PET/CT for BRPC than conventional imaging, particularly at low PSA levels, currently the best evidenced role for PSMA-PET/CT is restaging patients with BRPC. Given its high efficacy for detection of lymphatic metastases, PSMA-PET/CT may also become the standard of care in lymph node staging and preoperative planning prior to lymph node dissection. Other potential applications of PSMA-PET/CT currently under investigation include identification of the suspected primary site of PCa, primary staging of intermediate-to-high risk PCa, and targeted biopsy [42,43,44,45]. 

Preliminary evidence suggests that PSMA-targeted imaging significantly affects clinical practice. In a study of 118 patients with BRPC and high-risk (HR) PCa who underwent ^68^Ga-THP-PSMA PET/CT at diagnosis, management changed in 34% of patients (9/26) in the BRPC group and in 24% of patients (12/50) in the HR group [46]. In a prospective Phase II/III study of initial staging with ^18^F-DCFPyL PET/CT of 252 men with HR PCa who were planned for radical prostatectomy with lymphadenectomy, Pouliot et al. observed a PPV of 86.7% and reported that 22% of men (56/252) were upstaged to N1 or M1 disease by ^18^F-DCFPyL PET/CT [47]. PSMA-PET/CT may also better stratify patients potentially eligible for early SRT, given that SRT is commonly initiated in patients with serum PSA levels below those at which conventional imaging is reliably sensitive. Thus, more accurate localization of target volumes prior to SRT initiation might improve clinical response and reduce off-target effects. The impact of ^68^Ga-PSMA-11 PET/CT on the success rate of SRT for recurrent PCa after prostatectomy is currently being evaluated in a large randomized prospective trial (PSMA-SRT, NCT03582774) [48]. PSMA-PET/CT may also effectively identify patients for treatment with PSMA radioligand therapy (RLT). In a prospective Phase II trial that evaluated the efficacy of PSMA-PET/CT in predicting response to RLT, PSMA-PET/CT reliably predicted ≥30% PSA reduction, but no imaging parameters predicted ≥50% PSA reduction [49].

Improved localization of metastatic PCa using PSMA-targeted imaging may also increase the success rate of metastasis-directed therapy (MDT), including stereotactic body radiotherapy (SABR). MDT intends to postpone systemic treatment for patients with oligometastatic disease, thereby reducing the side-effects of hormonal therapy. Prospective data suggest that SABR is well tolerated and improves PFS in patients with oligometastatic PCa [50]. In the randomized STOMP study, which used choline PET/CT, stereotactic ablation of oligometastatic disease in 62 patients delayed the need for hormonal therapy from 13 to 21 months compared with surveillance [51]. Data from ORIOLE, a similar randomized Phase II study investigating the efficacy of SABR in forestalling metastases for hormone-sensitive PCa compared with observation, provide evidence for the value of PSMA PET/CT in controlling disease [52]. Patients randomized to the SABR arm of ORIOLE underwent ^18^F-DCFPyl PET/CT, a urea-based PSMA radiotracer, prior to and 180 days after treatment. Patients with no additional untreated lesions detected by PSMA PET/CT at baseline were significantly less likely to develop new metastatic lesions at six months than those whose PSMA PET/CT showed at least one additional lesion at baseline (16% vs. 63%, respectively).

Currently, the only PSMA-targeted imaging agent approved by the U.S. Food and Drug Administration is ^111^In-capromab pendetide (ProstaScint), which is approved for SPECT imaging of biopsy-proven PCa localized to the prostatic bed but at high risk for pelvic LNM. Regulatory approval of PSMA radiotracers in the United States has lagged behind other areas of the world, such as Europe and Australia, where much of the innovation in PSMA theranostics has occurred. Given the growing evidence of the clinical potential of PSMA, its availability is expanding in the United States, and regulatory approval of novel radiotracers, including ^68^Ga-PSMA-11 and ^18^F-DCFPyl, is expected within the next year. Ongoing clinical trials will help to better define the clinical role and impact of PSMA imaging and possibly strengthen the case for regulatory approval (Table 1). In sum, PSMA-targeted imaging has demonstrated clinical benefits through targeting stereotactic ablation in oligometastatic disease, but no studies have yet shown that PSMA-targeted imaging improves clinical outcomes for biochemically recurrent PCa patients.

## 4. PSMA Radioligand Therapy (RLT)

PSMA has also emerged as a promising therapeutic molecular target. Although various therapies are now approved for mCRPC, their survival benefit is generally limited to less than 6 months. There is thus a clinical need for novel therapies leading to a sustained response. Progress in the development of synthetic PSMA radioligands has led to an emerging body of research indicating significant therapeutic efficacy of PSMA RLT.

PSMA RLT studies have mostly utilized small-molecule inhibitors of PSMA as radioligands, which have been shown to be less hematotoxic than monoclonal antibodies. For instance, a study of MEDI3726 (a PSMA-targeted antibody–drug conjugate) in patients with mCRPC after failure of abiraterone or enzalumatide observed significant responses at higher doses of MEDI3726, although responses were not durable because patients discontinued therapy due to drug-related adverse events [53]. Small-molecule PSMA inhibitors have been labeled with both beta- and alpha-emitting radioisotopes, which have variable energy levels and path lengths. Beta-emitting radioisotopes, such as lutetium-177 (^177^Lu), are the favored radioisotopes given their short maximal tissue penetration and relatively long half-life, permitting delivery of a high degree of radiation to PCa lesions. Advantages of alpha-emitting radioisotopes include reduced red-marrow infiltration, leading to less hematotoxicity. In a proof-of-concept paper, application of the alpha-emitting ^225^actinium (^225^Ac) to two patients with diffuse bone marrow involvement led to undetectable PSA levels in both patients and no relevant hematotoxicity [54].

Among alpha-emitting radioisotopes, preliminary investigations of ^225^Ac-PSMA-617 suggest that it has substantial therapeutic efficacy for mCRPC. In a study of 17 chemotherapy-naive patients with advanced metastatic PCa treated with ^225^Ac-PSMA-617, 82% of patients had a PSA decline of ≥90%, and 41% of patients had undetectable serum PSA 12 months after therapy [55]. Targeted alpha-therapy may also benefit patients resistant to beta-emitting therapy and patients for whom beta-emitting therapy is contraindicated [54].

Among beta-emitting radioisotopes, ^177^Lu-PSMA RLT has been extensively studied and has consistently demonstrated a substantial decline in PSA in mCRPC patients. Among radioligands, ^177^Lu-PSMA-I&T and ^177^Lu-PSMA-617 are the most commonly utilized, but ^177^Lu-PSMA-617 has the preferred pharmacokinetic profile due to reduced kidney uptake [56]. More than 10 studies with small patient cohorts have observed significant PSA declines in patients with mCRPC treated with ^177^Lu-PSMA RLT (either ^177^Lu-PSMA-617 or ^177^Lu-PSMA-I&T) [57,58,59,60,61,62,63,64,65,66,67]. In a meta-analysis of ^177^Lu-PSMA-617 and ^177^Lu-PSMA-I&T studies, the pooled proportions of patients with any PSA decline and a ≥50% PSA decline were 71% (165/238) and 51% (123/238), respectively [68]. In a large cohort study of ^177^Lu-PSMA-617, investigators reported some PSA decline in 60% of patients and a PSA decline ≥50% in 45% of patients [69]. No head-to-head study of ^177^Lu-PSMA-617 and ^177^Lu-PSMA-I&T has been conducted. ^177^Lu-PSMA-617 is also currently being investigated in a large Phase III study (VISION, NCT 03511664), which is testing ^177^Lu-PSMA-617 in addition to standard of care vs. standard of care in patients with mCRPC previously exposed to at least one chemotherapy and one novel hormonal agent. This study is expected to read out in 2021.

The few studies that have examined overall survival (OS) of ^177^Lu-PSMA RLT have observed OS rates comparable to currently available third-line therapies. In a cohort of 59 patients with CRPC who were previously treated with second-generation ADT and chemotherapy, Brauer et al. reported a median progression-free survival (PFS) of 4.5 months and median OS of 8 months [59]. A retrospective study with a larger (104 patients) and more homogenous cohort of patients who were heavily pretreated reported a median OS of 14 months [70]. Investigators determined that a decline of at least 20.87% was the optimal parameter for predicting improved OS, but no specific level of PSA decline has been established as a surrogate for OS. Any PSA decline after the first cycle of ^177^Lu-RLT has been reported as a significant prognosticator of survival [59,71]. In the first prospective Phase II study of 30 patients with CRPC, investigators reported median PSA PFS and OS of 7.6 months and 13.5 months, respectively [63]. Compared to conventional third-line therapies for CRPC, a recent systematic review found that median OS was longer with ^177^Lu-PSMA RLT than with third-line treatment, but the difference was not statistically significant (mean of 14 months vs. 12 months, respectively, *p* = 0.32) [72].

Inter- and intra-patient heterogeneity of PSMA expression has been cited as a potential pitfall of PSMA RLT and may limit its clinical application. Studies of primary PCa suggest high homogeneity of PSMA expression [73]. However, immunohistochemistry studies of mCRPC lesions have noted significant inter- and intra-patient heterogeneity of PSMA expression [7,74]. Preclinical research has suggested that despite an overall increase in PSMA expression during progression of PCa from androgen sensitivity to androgen independence, some metastatic cell lines lose PSMA expression [75]. A significant proportion of liver metastases may also lack PSMA expression, although most liver metastases highly overexpress PSMA [76]. Heterogeneity of PSMA expression may partly explain why about 30% of patients do not respond to ^177^Lu-PSMA RLT [77]. In contrast, low PSMA expression in patients with mCRPC who progress after conventional therapies may be a negative prognostic indicator [78].

PSMA has also been the subject of novel immunotherapeutic approaches to mCRPC, such as bispecific T-cell engagers (BiTEs). BiTEs are a class of novel antibodies that form a link between T cells and tumor cells, permitting T-cell cytotoxic activity and initiating apoptosis of malignant cells. The PSMA/CD3-bispecific BiTE antibody BAY2010112 (AMG212, MT112) has been found to potently suppress tumor growth in preclinical research and was found to have an acceptable safety profile and dose-dependent clinical activity in a Phase I study of 16 patients [79,80].

### Safety of PSMA RLT

Studies have reported encouraging results on the safety of PSMA RLT. In the aforementioned multicenter study of 145 patients with mCRPC treated with ^177^Lu-PSMA-617, Grade 3 to 4 toxicities including anemia, leukopenia, and thrombocytopenia were reported in 10%, 3%, and 4% of patients, respectively [69]. Salivary gland toxicity, including mild or transient xerostomia, occurred in 8% of patients. A similar safety profile was reported in a study of 49 patients treated with three cycles of ^177^Lu-PSMA-617, but no Grade 4 hematotoxicity was observed and there were no significant differences between the PSMA RLT group and the control group in incidence of leukopenia or thrombocytopenia [71]. Mild nausea, loss of appetite, and fatigue are the most common nonhematologic adverse effects reported in studies of ^177^Lu PSMA-617. Despite the renal binding of PSMA ligands, research suggests ^177^Lu-PSMA-617 is relatively non-nephrotoxic. Low-grade nephrotoxicity has been reported, but there have been no reports of Grade 3 or 4 toxicity [81,82]. Risk factors for nephrotoxicity with ^177^Lu-PSMA-617 have also been identified. They include age (*p* = 0.049), hypertension (*p* = 0.001), and pre-existing kidney disease (*p* = 0.001) [81].

## 5. Conclusions

Extensive research has demonstrated the excellent diagnostic accuracy of PSMA-targeted imaging for the detection of BRPC, but the impact on overall survival of earlier initiation of therapy based on PSMA-targeted imaging has not yet been elucidated. Research is ongoing to define PSMA-targeted imaging’s exact role in other stages of the disease. Preliminary efficacy and safety data on PSMA RLT are very encouraging, and confirmatory data from larger studies will read out soon. While no PSMA RLT agent has yet obtained regulatory approval, federal approval is expected in the near future as ongoing studies read out (Table 2). The future of PSMA therapeutics may include novel radioisotopes, immunotherapeutic ligands, and combined approaches.

## Figures and Tables

**Table 1:** Ongoing Phase II and III clinical trials of PSMA-based imaging with enrolment of at least 100 participants.

Radioligand	Clinical Trial Identifier	Study Title	Phase	Primary Study Objective
^68^Ga-PSMA-11	NCT03362359	^68^Ga-PSMA-11 in High-risk Prostate Cancer	I/II	Assess safety and diagnostic performance of ^68^Ga-PSMA-11 PET/CT in patients with newly diagnosed high-risk PCa.
	NCT03439033	Comparison Study of PET/CT or PET/MRI Imaging to Magnetic Resonance Imaging (MRI) Alone in Men with Prostate Cancer	II	Compare diagnostic performance of ^68^Ga-PSMA-HBED-CC PET/CT and PET/MRI vs. MRI alone for primary Pca or BRCP.
	NCT04050215	^68^GA-PSMA-11 PET/CT Scan in Impacting Treatment Strategies for Patients with Prostate Cancer	II	Evaluate impact of ^68^Ga-PSMA-11 PET/CT on treatment strategy for BRPC.
	NCT03204123	PSMA PET Imaging of Recurrent Prostate Cancer	II	Evaluate diagnostic efficacy of ^68^Ga-HBED-CC-PSMA in BRPC.
	NCT03396874	^68^Ga-PSMA-11 PET in Patients with Biochemical Recurrence	II	Evaluate diagnostic performance of ^68^Ga-PSMA-11 PET/CT in BRPC.
	NCT03768349	^68^Ga-PSMA-11 and C-11 Choline PET in Patients with Biochemical Recurrence of Prostate Cancer	II	Evaluate diagnostic efficacy of ^68^Ga-PSMA-11 and C-11 Choline PET in metastatic PCa.
	NCT03689582	Radiolabeled ^68^Ga-PSMA for PET/CT Imaging to Detect Prostate Cancer	II	Evaluate diagnostic efficacy of ^68^Ga-PSMA-11 for the detection of primary PCa.
	NCT03762759	Fluciclovine F18 or ^68^Ga-PSMA PET/CT to Enhance Prostate Cancer Outcomes	II	Compare efficacy of Fluciclovine F18 or ^68^Ga--PSMA PET/CT in planning radiation treatments and enhancing outcomes in patients with PCa.
	NCT03822845	Evaluating the Clinical Accuracy of ^68^Ga-PSMA PET/CT Imaging in Patients with Biochemical Recurrence of Prostate Cancer	II/III	Evaluate diagnostic performance of ^68^Ga-PSMA PET/CT in BRPC.
	NCT02678351	^68^Ga-PSMA-11 PET/MRI in Finding Tumors in Patients with Intermediate or High-Risk Prostate Cancer Undergoing Surgery	II/III	Evaluate diagnostic performance of ^68^Ga-PSMA PET/MRI for detection of regional nodal and distant metastases in patients with intermediate- and high-risk PCa.
	NCT03001869	^68^Ga-PSMA PET/CT in Prostate Cancer	III	Determine safety, sensitivity, and specificity of ^68^Ga-PSMA PET/CT for BRPC.
	NCT02659527	PET/MRI in Patients With Suspected Prostate Cancer	III	Evaluate superiority of image guided biopsy using PSMA-PET/MRI in the diagnosis of primary PCa compared to conventional biopsy.
	NCT03582774	Trial of ^68^Ga-PSMA-11 PET/CT Molecular Imaging for Prostate Cancer Salvage Radiotherapy Planning (PSMA-SRT)	III	Evaluate the success rate of salvage radiation therapy (SRT) for recurrence of PCa after prostatectomy with and without planning based on ^68^Ga-PSMA-11 PET/CT.
	NCT03803475	^68^Ga-PSMA-11 PET Imaging in Prostate Cancer Patients	III	Evaluate diagnostic performance of ^68^Ga-PSMA-11 in detection of metastatic PCa.
	NCT03353740	^68^Ga-PSMA-11 Positron Emission Tomography (PET) Imaging in Patients with Biochemical Recurrence	III	Evaluate diagnostic performance of ^68^Ga-PSMA-11 in detection of BRPC.
	NCT03911310	^18^F-PSMA-11 PET/CT Phase 3 Clinical Study (NGP3)	III	Compare diagnostic performance of ^18^F-PSMA-11 and ^68^Ga-PSMA-11.
^18^F-DCFPyL	NCT03181867	^18^F-DCFPyL PET/CT in High Risk and Recurrent Prostate Cancer	II	Assess the ability of ^18^F-DCFPyL PET/CT to accurately stage high-risk primary PCa and detect sites of recurrent PCa.
	NCT03976843	Prostate Specific Membrane Antigen (PSMA)-Based PET Imaging of High Risk Prostate Cancer		Evaluate efficacy of ^18^F-DCFPyL in predicting recurrence of PCa in high-risk PCa prior to prostatectomy.
	NCT03471650	Study of PSMA-targeted ^18^F-DCFPyL PET/CT for the Detection of Clinically Significant Prostate Cancer	II	Evaluate diagnostic accuracy of ^18^F-DCFPyL PET/CT for detecting primary PCa.
	NCT03824275	^18^F-DCFPyL Positron Emission Tomography (PET)/Computed Tomography (CT) in Men With Prostate Cancer	II/III	Evaluate the efficacy of ^18^F-DCFPyL PET/CT as a predictive biomarker of response to therapy.
	NCT03525288	PSMA-PET Guided Radiotherapy (PSMA-PETgRT)	II/III	Compare cancer control outcomes of definitive radiotherapy informed by PSMA-PET with radiotherapy guided by conventional staging only.
	NCT03594760	PSMA-PET: Deep Radiomic Biomarkers of Progression and Response Prediction in Prostate Cancer	III	Acquire PSMA-PET data in patients with PCa who receive treatment and follow-up in order to enable the discovery of predictive imaging biomarkers through deep learning techniques.
	NCT03739684	Study of ^18^F-DCFPyL PET/CT Imaging in Patients with Suspected Recurrence of Prostate Cancer (CONDOR)	III	Evaluate diagnostic performance of ^18^F-DCFPyL PET/CT in patients with suspected BRPC and negative or equivocal findings on conventional imaging.
^18^F-PSMA-1007	NCT04102553	^18^F-PSMA-1007 Versus F-18-Fluorocholine PET in Patients with Biochemical Recurrence	III	Compare diagnostic performance of ^18^F-PSMA-1007 Versus F-18-Fluorocholine PET in patients with BRPC.
rhPSMA 7.3 (18F)	NCT04186819	Imaging Study to Investigate the Safety and Diagnostic Performance of rhPSMA 7.3 (18F) in Newly Diagnosed Prostate Cancer (LIGHTHOUSE)	III	Evaluate safety and diagnostic performance of radio-hybrid prostate-specific membrane antigen (rhPSMA) 7.3 (18F) PET ligand in men with newly diagnosed prostate cancer.
	NCT04186845	Imaging Study to Investigate Safety and Diagnostic Performance of rhPSMA 7.3 (18F) PET Ligand in Suspected Prostate Cancer Recurrence (SPOTLIGHT)	III	Evaluate safety and diagnostic performance of radio-hybrid prostate-specific membrane antigen (rhPSMA) 7.3 (18F) in BRPC.

**Table 2:** Ongoing clinical trials of PSMA RLT with enrolment of at least 50 participants.

Radioligand	Clinical Trial Identifier	Study Title	Phase	Primary Study Objective
I-131-1095	NCT03939689	Radiotherapy in Combination With Enzalutamide in Patients with Metastatic Castration-resistant Prostate Cancer Who Are Chemotherapy Naive and Have Progressed on Abiraterone (ARROW)	II	Evaluate the safety and efficacy of I-131-1095 in combination with Enzalutamide in patients with PSMA-avid mCRPC.
^177^Lu-PSMA-617	NCT03874884	^177^Lu-PSMA-617 Therapy and Olaparib in Patients with Metastatic Castration Resistant Prostate Cancer (LuPARP)	I	Evaluate safety and tolerability of Olaparib and ^177^Lutetium-PSMA in patients with mCRPC.
	NCT03545165	^177^Lu-J591 and ^177^Lu-PSMA-617 Combination for mCRPC	I	Determine the dose-limiting toxicity and maximum tolerated dose (MTD) of combined ^177^Lu-PSMA-J591 and ^177^Lu-PSMA-671 in patients with mCRPC.
	NCT03392428	A Trial of ^177^Lu-PSMA617 Theranostic Versus Cabazitaxel in Progressive Metastatic Castration Resistant Prostate Cancer	II	Determine the activity and safety of ^177^Lu-PSMA vs. cabazitaxel in men with progressive mCRPC.
	NCT03454750	Radiometabolic Therapy (RMT) with ^177^Lu-PSMA617 in Advanced Castration Resistant Prostate Cancer (CRPC) (LU-PSMA)	II	Evaluate efficacy and toxicity of radiometabolic therapy with ^177^LuPSMA-617 in advanced CRPC.
	NCT04343885	In Men With Metastatic Prostate Cancer, What is the Safety and Benefit of Lutetium-177 PSMA Radionuclide Treatment in Addition to Chemotherapy (UpFrontPSMA)	II	Compare the effectiveness of ^177^Lu-PSMA therapy followed by docetaxel chemotherapy vs. docetaxel chemotherapy alone in patients with newly-diagnosed high-volume metastatic hormone-naïve prostate cancer (mHNPC).
	NCT03392428	A Trial of ^177^Lu-PSMA-617 Theranostic Versus Cabazitaxel in Progressive Metastatic Castration Resistant Prostate Cancer (TheraP)	II	Determine the activity and safety of ^177^Lu-PSMA vs. cabazitaxel in men with progressive mCRPC.
	NCT03511664	Study of ^177^Lu-PSMA-617 in Metastatic Castrate-Resistant Prostate Cancer (VISION)	III	Compare OS in patients with progressive PSMA-positive mCRPC who receive ^177^Lu-PSMA-617 in addition to best supportive/best standard of care versus patients treated with best supportive/best standard of care alone.
^177^Lu-PSMA-R2	NCT03490838	^177^Lu-PSMA-R2 in Patients with PSMA Positive Progressive, Metastatic, Castration Resistant Prostate Cancer (PROter)	I/II	Assess safety, tolerability, radiation dosimetry, and preliminary efficacy of ^177^Lu-PSMA-R2 in patients with mCRPC.
^177^Lu-PSMA-J591	NCT00538668	Radioimmunotherapy in Prostate Cancer Using ^177^Lu-J591 Antibody	I	Determine the highest possible safe dose of ^177^Lu-PSMA-J591 and assess its effects on PCa.
	NCT03545165	^177^Lu-J591 and ^177^Lu-PSMA-617 Combination of mCRPC	I/II	Determine dose-limiting toxicity and MTD of the combination of ^177^Lu-J591 and ^177^Lu-PSMA-617.
AMG 160	NCT03792841	A Phase 1 Study Evaluating the Safety, Tolerability, Pharmacokinetics, and Efficacy of Prostate Specific Membrane Antigen Half-life Extended Bispecific T-cell Engager AMG 160 in Subjects With Metastatic Castration-resistant Prostate Cancer	I	Evaluate the safety and tolerability of AMG 160, a half-life-extended (HLE) bispecific T-cell engager (BiTE^®^) antibody.
BAY 2315497	NCT03724747	Study to Evaluate the Safety, Tolerability, Pharmacokinetics, and Anti-tumor Activity of a Thorium-227 Labeled Antibody-chelator Conjugate, in Patients with Metastatic Castration Resistant Prostate Cancer	I	Define the safety and tolerability profile and MTD of BAY 2315497.
AMG 509	NCT04221542	Study of AMG 509 in Subjects with Metastatic Castration-Resistant Prostate Cancer	I	Evaluate the safety, tolerability, and MTD of AMG 509 in adult subjects.

## References

[1] Wright G.L., Haley C., Beckett M.L., Schellhammer P.F. (1995). Expression of prostate-specific membrane antigen in normal, benign, and malignant prostate tissues. Urol. Oncol..

[2] Silver D.A., Pellicer I., Fair W.R., Heston D.W., Cordon-Cardo C. (1997). Prostate-specific membrane antigen expression in normal and malignant human tissues. Clin. Cancer Res..

[3] Minner S., Wittmer C., Graefen M., Salomon G., Steuber T., Haese A., Huland H., Bokemeyer C., Yekebas E., Dierlamm J. (2011). High level PSMA expression is associated with early PSA recurrence in surgically treated prostate cancer. Prostate.

[4] Bakht M.K., Derecichei I., Li Y., Ferraiuolo R., Dunning M., Oh S.W., Hussein A., Youn H., Stringer K.F., Jeong C.W. (2018). Neuroendocrine differentiation of prostate cancer leads to PSMA suppression. Endocr. Relat. Cancer.

[5] Mhawech-Fauceglia P., Zhang S., Terracciano L., Sauter G., Chadhuri A., Herrmann F.R., Penetrante R. (2007). Prostate-specific membrane antigen (PSMA) protein expression in normal and neoplastic tissues and its sensitivity and specificity in prostate adenocarcinoma: An immunohistochemical study using multiple tumour tissue microarray technique. Histopathology.

[6] Sweat S.D., Pacelli A., Murphy G.P., Bostwick D.G. (1998). Prostate-specific membrane antigen expression is greatest in prostate adenocarcinoma and lymph node metastases. Urology.

[7] Mannweiler S., Amersdorfer P., Trajanoski S., Terrett J.A., King D., Mehes G. (2009). Heterogeneity of prostate-specific membrane antigen (PSMA) expression in prostate carcinoma with distant metastasis. Pathol. Oncol. Res..

[8] Grubmuller B., Baum R.P., Capasso E., Singh A., Ahmadi Y., Knoll P., Floth A., Righi S., Zandieh S., Meleddu C. (2016). (64)Cu-PSMA-617 PET/CT Imaging of Prostate Adenocarcinoma: First In-Human Studies. Cancer Biother. Radiopharm..

[9] Afshar-Oromieh A., Malcher A., Eder M., Eisenhut M., Linhart H.G., Hadaschik B.A., Holland-Letz T., Giesel F.L., Kratochwil C., Haufe S. (2013). PET imaging with a [68Ga]gallium-labelled PSMA ligand for the diagnosis of prostate cancer: Biodistribution in humans and first evaluation of tumour lesions. Eur. J. Nucl. Med. Mol. Imaging.

[10] Calabria F., Pichler R., Leporace M., Wolfsgruber J., Coscarelli P., Dunzinger A., Schillaci O., Cascini G.L., Bagnato A. (2019). 68Ga/64Cu PSMA Bio-Distribution in Prostate Cancer Patients: Potential Pitfalls for Different Tracers. Curr. Radiopharm..

[11] Demirci E., Sahin O.E., Ocak M., Akovali B., Nematyazar J., Kabasakal L. (2016). Normal distribution pattern and physiological variants of 68Ga-PSMA-11 PET/CT imaging. Nucl. Med. Commun..

[12] Hovels A.M., Heesakkers R.A.M., Adang E.M., Strum S., Hoogeveen Y.L., Severens J.L., Barentsz J.O. (2008). The diagnostic accuracy of CT and MRI in the staging of pelvic lymph nodes in patients with prostate cancer: A meta-analysis. Clin. Radiol..

[13] Moffett S., Melancon D., DeCrescenzo G., St-Pierre C., Deschenes F., Saragovi H.U., Gold P., Cuello A.C. (2007). Preparation and characterization of new anti-PSMA monoclonal antilbodies with potential clinical use. Hybridoma.

[14] Nanus D.M., Milowsky M.I., Kostakoglu L., Smith-Jones P.M., Vallabahajosula S., Goldsmith S.J., Bander N.H. (2003). Clinical use of monoclonal antibody Hu-J591 therapy; targeting of prostate specific membrane antigen. J. Urol..

[15] Tan N., Bavadian N., Calais J., Oyoyo U., Kim J., Turkbey I.B., Mena E., Davenoport M.S. (2019). Imaging of Prostate Specific Membrane Antigen Targeted Radiotracers for the Detection of Prostate Cancer Biochemical Recurrence after Definitive Therapy: A Systematic Review and Meta-Analysis. J. Urol..

[16] Fendler W.P., Calais J., Eiber M., Flavell R.R., Mishoe A., Feng F.Y., Nguyen H.G., Reiter R.E., Rettig M.B., Okamoto S. (2019). Assessment of 68Ga-PSMA-11 PET Accuracy in Localizing Recurrent Prostate Cancer: A Prospective Single-Arm Clinical Trial. JAMA Oncol..

[17] Morigi J.J., Stricker P.D., Leeuwen P.J., Tang R., Ho B., Nguyen Q., Hruby G., Fogarty G., Jagavkar R., Kneebone A. (2015). Prospective Comparison of 18F-Fluoromethylcholine Versus 68Ga-PSMA PET/CT in Prostate Cancer Patients Who Have Rising PSA After Curative Treatment and Are Being Considered for Targeted Therapy. J. Nucl. Med..

[18] Bluemel C., Krebs M., Polat B., Linke F., Eiber M., Samnick S., Lapa C., Lassmann M., Riedmiller H., Czernin J. (2016). 68Ga-PSMA-PET/CT in Patients With Biochemical Prostate Cancer Recurrence and Negative 18F-Choline-PET/CT. Clin. Nucl. Med..

[19] Calais J., Ceci F., Eiber M., Hope T.A., Hofman M.S., Rischpler C., Bach-Gansmo T., Nanni C., Savir-Baruch B., Elashoff D. (2019). 18F-fluciclovine PET-CT and 68Ga-PSMA-11 PET-CT in Patients With Early Biochemical Recurrence After Prostatectomy; A Prospective, Single-Centre, Single-Arm, Comparative Imaging Trial. Lancet Oncol..

[20] Pernthaler B., Kulnik R., Gstettner C., Salamon S., Aigner R.M., Kvaternik H. (2019). A Prospective Head-to-Head Comparison of 18F-Fluciclovine With 68Ga-PSMA-11 in Biochemical Recurrence of Prostate Cancer in PET/CT. Clin. Nucl. Med..

[21] Wondergem M., Jansen B.H.E., van der Zant F.M., van der Sluis T.M., Knol R.J.J., van Kalmthout L.W.M., Hoekstra O.S., van Moorselaar R.J.A., Oprea-lager D.E., Vis A.N. (2019). Early lesion detection with (18)F-DCFPyL PET/CT in 248 patients with biochemically recurrent prostate cancer. Eur. J. Nucl. Med. Mol. Imaging.

[22] Giesel F.L., Knorr K., Spohn F., Will L., Maurer T., Flechsig P., Neels O., Schiller K., Amaral H., Weber W.A. (2019). Detection Efficacy of (18)F-PSMA-1007 PET/CT in 251 Patients with Biochemical Recurrence of Prostate Cancer After Radical Prostatectomy. J. Nucl. Med..

[23] Kuten J., Fahoum I., Savin Z., Shamni O., Gitstein G., Hershkovitz D., Mabjeesh N.J., Yossepowitch O., Mishani E., Even-Sapir E. (2019). Head- to head Comparison of (68)Ga-PSMA-11 with (18)F-PSMA-1007 PET/CT in Staging Prostate Cancer Using Histopathology and Immunohistochemical Analysis as Reference-Standard. J. Nucl. Med..

[24] Petersen L.J., Nielsen J.B., Langkilde N.C., Petersen A., Afshar-Oromieh A., De Souza N.M., De Paepe K., Fisker R.V., Arp D.T., Carl J. (2019). (68)Ga-PSMA PET/CT compared with MRI/CT and diffusion-weighted MRI for primary lymph node staging prior to definitive radiotherapy in prostate cancer: A prospective diagnostic test accuracy study. World J. Urol..

[25] Pfister D., Porres D., Heidenreich A., Heidegger I., Knuechel R., Steib F., Behrendt F.F., Verburg F.A. (2016). Detection of recurrent prostate cancer lesions before salvage lymphadenectomy is more accurate with (68)Ga-PSMA-HBED-CC than with (18)F-Fluoroethylcholine PET/CT. Eur. J. Nucl. Med. Mol. Imaging.

[26] Abufaraj M., Grubmuller B., Zeitlinger M., Kramer G., Seitz C., Haitel A., Baltzer P., Hacker M., Wadsak W., Pfaff S. (2019). Prospective evaluation of the performance of [(68)Ga]Ga-PSMA-11 PET/CT(MRI) for lymph node staging in patients undergoing superextended salvage lymph node dissection after radical prostatectomy. Eur. J. Nucl. Med. Mol. Imaging.

[27] Maurer T., Gschwend J.E., Rauscher I., Souvatzoglou M., Haller B., Weirich G., Wester H.J., Heck M., Kubler H., Beer A.J. (2016). Diagnostic Efficacy of (68)Gallium-PSMA Positron Emission Tomography Compared to Conventional Imaging for Lymph Node Staging of 130 Consecutive Patients with Intermediate to High Risk Prostate Cancer. J. Urol..

[28] Cantiello F., Gangemi V., Cascini G.L., Calabria F., Moschini M., Ferro M., Musi G., Buttice S., Salonia A., Briganti A. (2017). Diagnostic Accuracy of (64)Copper Prostate-specific Membrane Antigen Positron Emission Tomography/Computed Tomography for Primary Lymph Node Staging of Intermediate- to High-risk Prostate Cancer: Our Preliminary Experience. Urology.

[29] Budaus L., Leyh-Bannurah S., Salomon G., Michl U., Heinzer H., Huland H., Graefen M., Steuber T., Rosenbaum C. (2016). Initial Experience of (68)Ga-PSMA PET/CT Imaging in High-risk Prostate Cancer Patients Prior to Radical Prostatectomy. Eur. Urol..

[30] Zacho H.D., Nielsen J.B., Haberkorn U., Stenholt L., Petersen L.J. (2017). (68) Ga-PSMA PET/CT for the detection of bone metastases in prostate cancer: A systematic review of the published literature. Clin. Physiol. Funct. Imaging.

[31] Dadgar H., Emami F., Norouzbeigi N., Vafaee M.S., Jafari E., Gholamrezanezhad A., Assadi M., Amadzadehfar H. (2019). Application of [(68)Ga]PSMA PET/CT in Diagnosis and Management of Prostate Cancer Patients. Mol. Imaging Biol..

[32] Afshar-Oromieh A., Holland-letz T., Giesel F.L., Kratochwil C., Mier W., Haufe S., Debus N., Eder M., Eisenhut M., Schafer M. (2017). Diagnostic performance of (68)Ga-PSMA-11 (HBED-CC) PET/CT in patients with recurrent prostate cancer: Evaluation in 1007 patients. Eur. J. Nucl. Med. Mol. Imaging.

[33] Eiber M., Maurer T., Souvatzoglou M., Beer A.J., Ruffani A., Haller B., Graner F., Kubler H., Haberkorn U., Eisenhut M. (2015). Evaluation of Hybrid (6)(8)Ga-PSMA Ligand PET/CT in 248 Patients with Biochemical Recurrence After Radical Prostatectomy. J. Nucl. Med..

[34] Israeli R.S., Powell C.T., Fair W.R., Heston W.D. (1994). Expression of the prostate-specific membrane antigen. Cancer Res..

[35] Wright G.L., Grob B.M., Haley C., Grossman K., Newhall K., Petrylak D., Troyer J., Konchuba A., Schellhammer P.F., Moriarty R. (1996). Upregulation of prostate-specific membrane antigen after androgen-deprivation therapy. Urology.

[36] Evans M.J., Smith-Jones P.M., Wongvipat J., Navarro V., Kim S., Bander N.H., Larson S.M., Sawyers C.L. (2011). Noninvasive measurement of androgen receptor signaling with a positron-emitting radiopharmaceutical that targets prostate-specific membrane antigen. Proc. Natl. Acad. Sci. USA.

[37] Hope T.A., Truillet C., Ehman E.C., Afshar-Oromieh A., Aggarwal R., Ryan C.J., Carroll P.R., Small E.J., Evans M.J. (2017). 68Ga-PSMA-11 PET Imaging of Response to Androgen Receptor Inhibition: First Human Experience. J. Nucl. Med..

[38] Afshar-Oromieh A., Hetzheim H., Kratochwil C., Benesova M., Eder M., Neels O.C., Eisenhut M., Kubler W., Holland-Letz T., Giesel F.L. (2015). The Theranostic PSMA Ligand PSMA-617 in the Diagnosis of Prostate Cancer by PET/CT: Biodistribution in Humans, Radiation Dosimetry, and First Evaluation of Tumor Lesions. J. Nucl. Med..

[39] Leitsmann C., Thelen P., Schmid M., Meller J., Sahlmann C., Meller B., Trojan L., Strauss A. (2019). Enhancing PSMA-uptake with androgen deprivation therapy a new way to detect prostate cancer metastases?. Int. Braz. J. Urol..

[40] Artigas C., Alexiou J., Garcia C., Wimana Z., Otte F., Van Velthoven R., Flamen P. (2016). Paget bone disease demonstrated on (68)Ga-PSMA ligand PET/CT. Eur. J. Nucl. Med. Mol. Imaging.

[41] Ardies P.J., Gykiere P., Goethals L., De Mey J., De Geeter F., Everaert H. (2017). PSMA Uptake in Mediastinal Sarcoidosis. Clin. Nucl. Med..

[42] Fendler W.P., Schmidt D.F., Wenter V., Thierfelder K.M., Zach C., Stief C., Bartenstein P., Kirchner T., Gildehaus F.J., Gratzke C. (2016). 68Ga-PSMA PET/CT Detects the Location and Extent of Primary Prostate Cancer. J. Nucl. Med..

[43] Yaxley J.W., Raveenthiran S., Nouhaud F., Samaratunga H., Yaxley W.J., Coughlin G., Yaxley A.J., Gianduzzo T., Kua B., McEwan L. (2019). Risk of metastatic disease on (68) gallium-prostate-specific membrane antigen positron emission tomography/computed tomography scan for primary staging of 1253 men at the diagnosis of prostate cancer. Bju. Int..

[44] Cytawa W., Seitz A.K., Kircher S., Fukushima K., Tran-Gia J., Schirbel A., Bandurski T., Lass P., Krebs M., Polom W. (2020). (68)Ga-PSMA I&T PET/CT for primary staging of prostate cancer. Eur. J. Nucl. Med. Mol. Imaging.

[45] Zhang J., Shao S., Wu P., Liu D., Yang B., Han D., Li Y., Lin X., Song W., Cao M. (2019). Diagnostic performance of (68)Ga-PSMA PET/CT in the detection of prostate cancer prior to initial biopsy: Comparison with cancer-predicting nomograms. Eur. J. Nucl. Med. Mol. Imaging.

[46] Kulkarni M., Hughes S., Mallia A., Gibson V., Young J., Aggarwal A., Morris S., Challacombe B., Popert R., Brown C. (2020). The management impact of (68)gallium-tris(hydroxypyridinone) prostate-specific membrane antigen ((68)Ga-THP-PSMA) PET-CT imaging for high-risk and biochemically recurrent prostate cancer. Eur. J. Nucl. Med. Mol. Imaging.

[47] Pouliot F., Carroll P., Probst S., Pienta K.J., Rowe S.P., Saperstein L., Siegel B., Patnaik A., Preston M.A., Alva A.S. (2020). A prospective phase II/III multicenter study of PSMA-targeted 18F-DCFPyL PET/CT imaging in patients with prostate cancer (OSPREY): A sub-analysis of regional and distant metastases detection rates at initial staging by 18F-DCFPyL PET/CT. J. Clin. Onc..

[48] Calais J., Czernin J., Fendler W.P., Elashoff D., Nickols N.G. (2019). Randomized prospective phase III trial of 68Ga-PSMA-11 PET/CT molecular imaging for prostate cancer salvage radiotherapy planning [PSMA-SRT]. BMC Cancer.

[49] Emmett L., Crumbaker M., Ho B., Willowson K., Eu P., Ratnayake L., Epstein R., Blanskby A., Horvath L., Guminski A. (2019). Results of a Prospective Phase 2 Pilot Trial of (177)Lu-PSMA-617 Therapy for Metastatic Castration-Resistant Prostate Cancer Including Imaging Predictors of Treatment Response and Patterns of Progression. Clin. Genitourin. Cancer..

[50] Siva S., Bressel M., Murphy D.G., Shaw M., Chander S., Violet J., Tai K.H., Udovicich C., Lim A., Selbie L. (2018). Stereotactic Abative Body Radiotherapy (SABR) for Oligometastatic Prostate Cancer: A Prospective Clinical Trial. Eur. Urol..

[51] Ost P., Reynders D., Decaestecker K., Fonteyne V., Lumen N., De Bruycker A., Lambert B., Delrue L., Bultijnck R., Claeys T. (2018). Surveillance or Metastasis-Directed Therapy for Oligometastatic Prostate Cancer Recurrence: A Prospective, Randomized, Multicenter Phase II Trial. J. Clin. Oncol..

[52] Phillips R., Lim S.J., Shi W.Y., Antonarakis E.S., Rowe S., Gorin M., Deville C., Greco S.C., Denmeade S., Paller C. (2019). Primary Outcomes of a Phase II Randomized Trial of Observation Versus Stereotactic Ablative RadiatIon for OLigometastatic Prostate CancEr (ORIOLE). Int. J. Radiat..

[53] De Bono J.S., Fleming M.T., Wang J.S., Cathomas R., Williams M., Bothos J.G., Balic K., Cho S.H., Martinez P., Petrylak D.P. (2020). MEDI3726, a prostate-specific membrane antigen (PSMA)-targeted antibody-drug conjugate (ADC) in mCRPC after failure of abiraterone or enzalutamide. J. Clin. Oncol..

[54] Kratochwil C., Bruchertseifer F., Giesel F.L., Weis M., Verburg F.A., Mottaghy F., Kopka K., Apostolidis C., Haberkorn U., Morgenstern A. (2016). 225Ac-PSMA-617 for PSMA-Targeted alpha-Radiation Therapy of Metastatic Castration-Resistant Prostate Cancer. J. Nucl. Med..

[55] Sathekge M., Bruchertseifer F., Knoesen O., Reyneke F., Lawal I., Lengana T., Davis C., Mahapane J., Corbett C., Vorster M. (2019). (225)Ac-PSMA-617 in chemotherapy-naive patients with advanced prostate cancer: A pilot study. Eur. J. Nucl. Med. Mol. Imaging.

[56] Benesova M., Schafer M., Bauder-Wust U., Afshar-Oromieh A., Kratochwil C., Mier W., Haberkorn U., Kopka K., Eder M. (2015). Preclinical Evaluation of a Tailor-Made DOTA-Conjugated PSMA Inhibitor with Optimized Linker Moiety for Imaging and Endoradiotherapy of Prostate Cancer. J. Nucl. Med..

[57] Yadav M.P., Ballal S., Tripathi M., Damle N.A., Sahoo R.K., Seth A., Bal C. (2017). (177)Lu-DKFZ-PSMA-617 therapy in metastatic castration resistant prostate cancer: Safety, efficacy, and quality of life assessment. Eur. J. Nucl. Med. Mol. Imaging.

[58] Scarpa L., Buxbaum S., Kendler D., Fink K., Bektic J., Gruber L., Decristoforo C., Uprimny C., Lukas P., Horninger W. (2017). The (68)Ga/(177)Lu theragnostic concept in PSMA targeting of castration-resistant prostate cancer: Correlation of SUVmax values and absorbed dose estimates. Eur. J. Nucl. Med. Mol. Imaging.

[59] Brauer A., Grubert L.S., Roll W., Schrader A.J., Schafers M., Bogermann M., Rahbar K. (2017). (177)Lu-PSMA-617 radioligand therapy and outcome in patients with metastasized castration-resistant prostate cancer. Eur. J. Nucl. Med. Mol. Imaging.

[60] Fendler W.P., Reinhardt S., Ilhan H., Delker A., Boning G., Gildehaus F.J., Stief C., Bartenstein P., Gratzke C., Lehner S. (2017). Preliminary experience with dosimetry, response and patient reported outcome after 177Lu-PSMA-617 therapy for metastatic castration-resistant prostate cancer. Oncotarget.

[61] Heck M.M., Tauber R., Schwaiger S., Retz M., D’Alessandria C., Maurer T., Gafita A., Wester H., Gschwend J.E., Weber W.A. (2019). Treatment Outcome, Toxicity, and Predictive Factors for Radioligand Therapy with (177)Lu-PSMA-I&T in Metastatic Castration-resistant Prostate Cancer. Eur. Urol..

[62] Baum R.P., Kulkarni H.R., Schuchardt C., Singh A., Wirtz M., Wiessalla S., Schottelius M., Mueller D., Klette I., Wester H. (2016). 177Lu-Labeled Prostate-Specific Membrane Antigen Radioligand Therapy of Metastatic Castration-Resistant Prostate Cancer: Safety and Efficacy. J. Nucl. Med..

[63] Hofman M.S., Violet J., Hicks R.J., Ferdinandus J., Thang S.P., Akhurst T., Iravani A., Kong G., Kumar A.R., Murphy D.G. (2018). [(177)Lu]-PSMA-617 radionuclide treatment in patients with metastatic castration-resistant prostate cancer (LuPSMA trial): A single-centre, single-arm, phase 2 study. Lancet Oncol..

[64] Kratochwil C., Giesel F.L., Stefanova M., Benesova M., Bronzel M., Afshar-Oromieh A., Mier W., Eder M., Kopka K., Haberkorn U. (2016). PSMA-Targeted Radionuclide Therapy of Metastatic Castration-Resistant Prostate Cancer with 177Lu-Labeled PSMA-617. J. Nucl. Med..

[65] Rahbar K., Bode A., Weckesser M., Avramovic N., Claesener M., Stegger L., Bogemann M. (2016). Radioligand Therapy With 177Lu-PSMA-617 as A Novel Therapeutic Option in Patients With Metastatic Castration Resistant Prostate Cancer. Clin. Nucl. Med..

[66] Ahmadzadehfar H., Rahbar K., Kurpig S., Bogemann M., Claesener M., Eppard E., Gartner F., Rogenhofer S., Schafers M., Essler M. (2015). Early side effects and first results of radioligand therapy with (177)Lu-DKFZ-617 PSMA of castrate-resistant metastatic prostate cancer: A two-centre study. Ejnmmi. Res..

[67] Ahmadzadehfar H., Eppard E., Kurpig S., Fimmers R., Yordanova A., Schlenkhoff C.D., Gartner F., Rogenhofer S., Essler M. (2016). Therapeutic response and side effects of repeated radioligand therapy with 177Lu-PSMA-DKFZ-617 of castrate-resistant metastatic prostate cancer. Oncotarget.

[68] Calopedos R.J.S., Chalasani V., Asher R., Emmett L., Woo H.H. (2017). Lutetium-177-labelled anti-prostate-specific membrane antigen antibody and ligands for the treatment of metastatic castrate-resistant prostate cancer: A systematic review and meta-analysis. Prostate. Cancer Prostatic. Dis..

[69] Rahbar K., Ahmadzadehfar H., Kratochwil C., Haberkorn U., Schafers M., Essler M., Baum R.P., Kulkarni H.R., Schmidt M., Drzezga A. (2017). German Multicenter Study Investigating 177Lu-PSMA-617 Radioligand Therapy in Advanced Prostate Cancer Patients. J. Nucl. Med..

[70] Rahbar K., Boegemann M., Yordanova A., Eveslage M., Schafers M., Essler M., Ahmadzadehfar H. (2018). PSMA targeted radioligandtherapy in metastatic castration resistant prostate cancer after chemotherapy, abiraterone and/or enzalutamide. A retrospective analysis of overall survival. Eur. J. Nucl. Med. Mol. Imaging.

[71] Ahmadzadehfar H., Zimbelmann S., Yordanova A., Fimmers R., Kurpig S., Eppard E., Gaertner F.C., Wei X., Hauser S., Essler M. (2017). Radioligand therapy of metastatic prostate cancer using (177)Lu-PSMA-617 after radiation exposure to (223)Ra-dichloride. Oncotarget.

[72] Von Eyben F.E., Roviello G., Kiljunen T., Uprimny C., Virgolini I., Kairemo K., Joensuu T. (2018). Third-line treatment and (177)Lu-PSMA radioligand therapy of metastatic castration-resistant prostate cancer: A systematic review. Eur. J. Nucl. Med. Mol. Imaging.

[73] Tsourlakis M.C., Klein F., Kluth M., Quaas A., Graefen M., Haese A., Simon R., Sauter G., Schlomm T., Minner S. (2015). PSMA expression is highly homogenous in primary prostate cancer. Appl. Immunohistochem. Mol. Morphol..

[74] Paschalis A., Sheehan B., Riisnaes R., Rodrigues D.N., Gurel B., Bertan C., Ferreira A., Lambros M.B.K., Seed G., Yuan W. (2019). Prostate-specific Membrane Antigen Heterogeneity and DNA Repair Defects in Prostate Cancer. Eur. Urol..

[75] Laidler P., Dulinska J., Lekka M., Lekki J. (2005). Expression of prostate specific membrane antigen in androgen-independent prostate cancer cell line PC-3. Arch. Biochem. Biophys..

[76] Damjanovic J., Janssen J., Prasad V., Diederichs G., Walter T., Brenner W., Makowski M.R. (2019). (68)Ga-PSMA-PET/CT for the evaluation of liver metastases in patients with prostate cancer. Cancer Imaging.

[77] Farolfi A., Gafita A., Calais J., Eiber M., Afshar-Oromieh A., Spohn F., Barbato F., Weber M., Ilhan H., Cervati V. (2019). _68_Ga-PSMA-11 PET Detects Residual Prostate Cancer after Prostatectomy in a Multicenter Retrospective Study. J. Urol..

[78] Thang S.P., Violet J., Sandhu S., Iravani A., Akhurst T., Kong G., Kumar A.R., Murphy D.G., Williams S.G., Hicks R.J. (2019). Poor Outcomes for Patients with Metastatic Castration-resistant Prostate Cancer with Low Prostate-specific Membrane Antigen (PSMA) Expression Deemed Ineligible for (177)Lu-labelled PSMA Radioligand Therapy. Eur. Urol. Oncol..

[79] Hummel H., Kufer P., Grullich C., Deschler-Baier B., Chatterjee M., Goebeler M., Miller K., De Santis M., Loidl W.C., Buck A. (2019). Phase 1 study of pasotuxizumab (BAY 2010112), a PSMA-targeting Bispecific T cell Engager (BiTE) immunotherapy for metastatic castration-resistant prostate cancer (mCRPC). J. Clin. Oncol..

[80] Bailis J., Deegen P., Thomas O., Bogner P., Wahl J., Liao M., Li S., Matthes K., Nagele V., Rau D. (2019). Preclinical evaluation of AMG 160, a next-generation bispecific T cell engager (BiTE) targeting the prostate-specific membrane antigen PSMA for metastatic castration-resistant prostate cancer (mCRPC). J. Clin. Oncol..

[81] Yordanova A., Becker A., Eppard E., Kurpig S., Fisang C., Feldmann G., Essler M., Ahmadzadehfar H. (2017). The impact of repeated cycles of radioligand therapy using [(177)Lu]Lu-PSMA-617 on renal function in patients with hormone refractory metastatic prostate cancer. Eur. J. Nucl. Med. Mol. Imaging.

[82] Zhang J., Kulkarni H.R., Singh A., Schuchardt C., Niepsch K., Langbein T., Baum R.P. (2019). (177)Lu-PSMA-617 Radioligand Therapy in Metastatic Castration-Resistant Prostate Cancer Patients with a Single Functioning Kidney. J. Nucl. Med..

